# Surface Electromyography in Clinical Practice. A Perspective From a Developing Country

**DOI:** 10.3389/fneur.2020.578829

**Published:** 2020-10-15

**Authors:** Hachi Manzur-Valdivia, Joel Alvarez-Ruf

**Affiliations:** ^1^Red de Salud Universidad Católica-Christus, Santiago, Chile; ^2^Laboratorio de Cognición y Comportamiento Sensoriomotor, Departamento de Kinesiología, Universidad Metropolitana de Ciencias de la Educación, Santiago, Chile; ^3^Laboratorio de Biomecánica Clínica, Facultad de Medicina Clínica Alemana, Universidad del Desarrollo, Carrera de Kinesiología, Santiago, Chile

**Keywords:** surface electromyography, neurorehabilitation, physiotherapy education, low-income countries, Chile, clinical training, electromyographic biofeedback

## Abstract

Surface electromyography (sEMG) has long been used in research, health care, and other fields such as ergonomics and brain-machine interfaces. In health care, sEMG has been employed to diagnose as well as to treat musculoskeletal disorders, pelvic floor dysfunction, and post-stroke motor deficits, among others. Despite the extensive literature on sEMG, the clinical community has not widely adopted it. We believe that in developing countries, such as Chile, this phenomenon may be explained by several interacting barriers. First, the socioeconomics of the country creates an environment where only high cost-effective treatments are routinely applied. Second, the majority of the sEMG literature on clinical applications has not extensively translated into decisive outcomes, which interferes with its applicability in low-income contexts. Third, clinical training on rehabilitation provides inadequate instruction on sEMG. And fourth, accessibility to equipment (i.e., affordability, availability, portability) may constitute another barrier, especially among developing countries. Here, we analyze socio-economic indicators of health care in Chile and comment on current literature about the use of sEMG in rehabilitation. Then we analyze the curricula of several physical therapy schools in Chile and report some estimations of the training on sEMG. Finally, we analyze the accessibility of some available sEMG devices and show that several match predefined criteria. We conclude that in developing countries, the insufficient use of sEMG in health might be explained by a shortage of evidence showing a crucial role in specific outcomes and the lack of training in rehabilitation-related careers, which interact with local socioeconomic factors that limit the application of these techniques.

## Introduction

Since the ′40s, surface electromyography (sEMG) has been used in a variety of settings, including motor-control research, education, health care, rehabilitation, ergonomics, and human-computer interfaces, among others (1). Unlike needle EMG, which has long been used in the assessment of neuromuscular disorders, sEMG is rarely employed in clinical and rehabilitation practice.

The literature on sEMG is extensive, and despite the total publications counting in the thousands, the number of papers devoted to clinical applications is considerably fewer. For example, a search in PubMed with the terms “(surface EMG[Title/Abstract]) OR (sEMG[Title/Abstract]) OR (surface electromyography[Title/Abstract])” leads to 8,270 records. Adding terms such as “physiotherapy,” “stroke,” “gait,” or “back pain” leads to 60, 376, 497, 124, and 521 records, respectively. This suggests an important gap between the total literature on sEMG and the part of it dedicated to clinical applications. This gap prevents the inclusion of sEMG applications into clinical practice guidelines (2–7).

In developing countries, such as Chile, high inequities in per-capita income determine the quality and opportunity of health care. Combined with a centralized distribution of high-complexity health centers and specialists, these factors create a scenario where only high-impact and cost-effective interventions are applied. Therefore, potentially useful but non-critical tools—such as sEMG—are usually left outside the clinical armamentarium.

The underrepresentation of sEMG in clinical guidelines determines that in rehabilitation careers, these topics are either not routinely taught, or maybe included in theoretical courses (i.e., movement control, muscle physiology) but not in clinical internships. These might be substantial reasons that explain why rehabilitation professionals do not routinely use sEMG in their practice (8).

Another barrier to the widespread use of sEMG in developing countries may simply be the accessibility (i.e., cost, availability, portability) of the current EMG devices. Thus, we explore and compare the characteristics of several devices to shed some light on this topic.

## Socioeconomic Aspects

In developing countries, such as Chile, there are substantial barriers that impact health care access. The high inequity in income distribution determines the opportunity and quality of health care. For example, the Gini index, which measures income-distribution inequality (0: perfect equity, to 100: perfect inequity), shows that Chile scores 44.4 (9), placing it at the percentile 84 worldwide.

Public spending on health is also low. The Domestic General Government Health Expenditure index (10) shows that in 2017 Chile spent 4.5% of its GDP in health, placing it in the percentile 74 worldwide. Other countries in the region such as Argentina and Brazil spent 6.6 and 4%, respectively. Compared to Sweden (9.2%), Finland (7.1%), and Norway (8.9%), our expenditure places us far from developed countries.

Additionally, high-complexity health care centers and specialists are located mostly in the capital (11). According to the health department of the Chilean government (www.minsal.cl), there are currently 23 high-complexity (type 1), and 37 medium-high (type 2) hospitals in the country. According to our 2017 census, the current population is 17.5 million people. Considering that roughly 80% of this population is cared for by the public system, this implies that each of these centers has to take care of about 230,000 people.

These complex and intermingled factors create a scenario where health policies favor mostly high-impact and cost-effective interventions. For example, for 80 high-prevalence pathologies, the chilean government warrants access to diagnostic tools and treatments for which there is sufficient evidence of cost-effectiveness (12). As a particular case, for the treatment of acute ischemic stroke our public health system provides state of the art treatment (2) in many centers. Although expensive, there is enough evidence supporting the investment of our limited resources in such cost-effective interventions.

## Translation of sEMG Literature into Clinical Guidelines

During the last three decades, evidence-based medicine (EBM) has encouraged the testing of many procedures and interventions that were routinely prescribed but not clinically proven (13). Based on the best available evidence, many clinical societies have produced guidelines that establish levels of recommendations for different interventions. Therefore those tests, protocols, or treatments that reliably lead to good outcomes are highly recommended over those whose applications do not provide a benefit or even harm (2, 14).

In the case of sEMG, a growing number of publications have explored its use in gait analysis (15, 16), muscle fatigue (17, 18), low-back pain (19, 20), muscle activity onset latency (21, 22), ankle instability (21), and techniques of analysis (23), just to name a few. Although sEMG is essential for the understanding of neuromuscular physiology and dysfunction, the scarcity of literature demonstrating that it is instrumental for reaching favorable clinical outcomes has prevented its general inclusion in EBM guidelines and might be one of the main barriers for its widespread use in clinical practice.

As we discussed earlier, developing countries, such as Chile, favor high cost-effective approaches, which poses considerable obstacles in applying potentially effective but unproven tools.

### sEMG as a Tool for Therapy: Pearls and Pitfalls

As we previously discussed, sEMG has been an essential tool to understand the neuromuscular system; nevertheless, it has also been long employed as a tool for therapy in the form of biofeedback (24) to treat a number of conditions. In dysphagia, it has been used as an adjunctive treatment to standard therapy, where it increases the displacement of hyoid and the laryngeal elevation, increases myoelectrical activity, and improves swallowing (25, 26). In post-stroke motor deficits, it has been employed in the rehabilitation of upper and lower extremities. In the upper extremity, when compared to standard therapy, it improves motor scores, but not independence scores (FIM) (27). In the lower extremity, it improves the range of motion and clinical scores of impairment, although it is not clearly superior to standard therapy alone (28). In cervical and shoulder pain, the telerehabilitation treatment with EMG-BF has been shown to be at least as effective as conventional therapy in reducing pain scores (29). It also has been explored in the context of sleep bruxism, where it showed that using EMG-BF during the day produced a decrease in the amplitude of myoelectrical activity of masticatory muscles during sleep, although the impact of this finding in clinical outcomes is not clear (30). In a pelvic floor musculature training and education program, the group receiving EMG-BF training was found to have a better quality of life. Nevertheless, the control group did not receive any therapy, which prevents obtaining stronger conclusions (31). In spinal cord injury, the use of EMG-BF as part of the rehabilitation protocol, leads to higher levels of muscle activation when requesting an elbow flexion. Also, patients reported higher levels of motivation during therapy and considered it as a useful and valuable tool (32).

One of the main difficulties with the application of sEMG in this context is that the pooled evidence does not offer reliable supporting results, which has been reflected in clinical guidelines and, therefore, in clinical practice.

For example, a 2007 meta-analysis of 13 studies on EMG-BF for post-stroke rehabilitation (33) showed that the analyzed evidence was not sufficient to conclude that it provided an extra benefit over standard therapy in the recovery of stroke patients. A 2014 meta-analysis examined the evidence of several physical-therapy interventions in the recovery of stroke (34). Among those interventions, it assessed EMG-BF in the context of upper and lower limb function and gait. Although there is a tendency for a positive effect, the pooled analysis revealed that it does not add to the standard therapy. These data have crystallized into clinical guidelines such as the Canadian “Evidence-Based Review of Stroke” (4) or the American Heart Association guidelines on stroke rehabilitation (2), which do not offer strong recommendations for the use of EMG-BF for stroke rehabilitation.

Reasons for the failure of these meta-analysis are explained by high study heterogeneity (33), small sample sizes (26–28), lack of electrode placement description, which may not correspond to current standards (35, 36); and finally, an inability to reach a certain level on therapy intensity, which is decisive for obtaining significant outcomes (37–40).

Despite being successfully used in several fields, there has been limited pooling of data or systematic reviews of EMG-BF for interventions. The lack of demonstrated effect size resulting from this is a barrier for the implementation of this tool in clinical use.

## sEMG Training in Chilean Physical Therapy Schools

Surface electromyography offers several benefits to rehabilitation professionals, nevertheless, its lack of widespread use may also be explained by insufficient training. To approach this question, we contacted 17 physical therapy (PT) schools, and eight sent us the curricula from their career. These account for 21.1% of the PT students of the country (4,292 of 20,306). As a means to approach the level of influence these schools exert in the local educational landscape, we report the national ranking of the schools' Universities (Table 1) (41). By using a python script, we searched for the keywords “electromiografía,” “instrumentación,” “EMG,” and “sEMG.” Candidate courses were manually checked by the authors and were only included if the keywords appeared in the contents but not under different headings, such as “bibliography” or “suggested readings.” None of the collected course programs mentioned the number of hours or credits devoted to each of the contents, thus, approaching the time spent on electromyography-related content was not possible.

**Table 1:** Summary results of a simple analysis of the presence of electromyography-related keywords (“Electromiografía,” “Instrumentación,” “bioinstrumentación,” “EMG,” and “sEMG”) in the curricula of eight PT careers.

Number of PT curriculums analyzed	8
Number of PT students in the analyzed schools/Number of nationwide PT students	4,292/20,306 (21.1%)
Percentile of the national ranking of the PT schools' Universities included in this analysis	A: 1.4, B: 22.5, C: 7.0, D: 3.5, E: 29.6, F: 26.1, G: 33.1, and H: 31.7
Distribution of the percentiles of national rankings of the PT schools' Universities included in this analysis	Whitin percentile 10:3 Whitin percentile 25:4 Whitin percentile 50:8
Number of PT schools where any of the keywords are mentioned in the curriculum	5
Number of PT students from the sampled schools exposed to any EMG-related content/Number of PT students in the analyzed schools	3,780/4,292 (88.1%)
Number of courses where any of the keywords were mentioned/Total number of courses	A: 1/29, B: 1/60, C: 1/38, D: 2/51, and E: 3/69
Number of introductory or theoretical courses where any of the keywords are mentioned/Total number of courses in the first 3 years	A: 1/14, B: 1/39, C: 1/24, D: 2/36, and E: 3/39
Number of clinical oriented courses and internship where any of the keywords are mentioned/Total number of clinical or internship courses	A: 0/15, B: 0/21, C: 0/14, D: 0/15, and E: 0/30

*PT, physical therapy. The letters in the third to fifth row refer to the curriculum of different schools. The number of students for the year 2019 was obtained from https://www.mifuturo.cl/bases-de-datos-de-matriculados/*.

We first counted the number of curricula in which at least one course mentioned any of the keywords. This simple approach revealed that five out of eight curricula met these criteria (Table 1). These curricula account for 3,780 students in the country.

The total number of courses where EMG contents are present might be a loose proxy for the amount of training on this technique. Accordingly, for each curriculum with at least one EMG course, we counted the number of courses in which any of the keywords above were present. This approach resulted in a mode of one course per career mentioning any of the keywords (Table 1).

Finally, during the internship, the PT student is placed in a real clinical environment that shapes the repertoire of techniques that he or she will use as a professional therapist. Hence, the presence of sEMG content on these clinical internships might be crucial for the use of this technique as a future PT. We found that none of the internships and clinical oriented courses mentioned any EMG-related content in their description (Table 1). Thus, sEMG training is not provided in all the PT schools, and is taught only in courses that take place during the first 3 years, but not during clinical internships (Table 1).

## EMG Devices

Considering all the barriers to successfully applying sEMG to the clinical practice, a shortage of accessible EMG devices may add another barrier to its use. Here, we consider accessibility as the combination of portability, affordability, and ease of use of a particular EMG device. We define these criteria as follows:

- Portability: the device has a small size (pocket size, or hand-held device size), can be easily carried to different locations, has internal batteries, and does not require a computer for operation.
- Affordable: considering that one of the main end-users of these systems may be the physical therapist, we defined this term based on the average monthly income before taxes (AMI) of a Chilean PT. We chose this parameter because, in their clinical practice, many PTs have to purchase their own equipment. The official statistics indicate that the 1st year after school, the AMI is U$740, and during the 5th year after graduation, it rises to U$1,330 (42). Therefore USD 1,000 seemed like a reasonable threshold.
- Ease of use: all the necessary elements (hardware, software) are provided, and is compatible with smartphones or tablets (obtained from brochures or website descriptions).

The results are described in Table 2. Some of the devices found are expensive and more suited for research (BTS, Noraxon, Delsys, and Bioelettronica). On the other hand, the most inexpensive one, the Myowave, requires buying additional hardware (i.e., an Arduino board) and programming skills. Thus it is not suited for immediate clinical use.

**Table 2:** Description of solutions.

**Company**	**Model**	**Cost**	**Link**	**Portability**	**Affordable**	**Ease of use**
Advancer Technologies	Myoware	USD 37.99	http://www.advancertechnologies.com/p/myoware.html	Yes	Yes	No (requires additional hardware)
EMG One	EMG One	USD 410	http://waves-tech.cl	Yes	Yes	Yes
Athos	Athos	USD 348	https://shop.liveathos.com/	Yes	Yes	Yes
Shimmer technology	Shimmer3 Consensys EMG Development Kit	USD 534	http://www.shimmersensing.com/products/emg-develop-kit	Yes	Yes	Yes
OT-Bioelettronica	Forza, Duelite	~USD 948–1,422	https://www.otbioelettronica.it/en/products/hardware/item/101-quattrocento-en	Yes	Yes ❖	Yes
mTrigger	mTrigger Biofeedback	USD 399–1.099	https://www.mtrigger.com/	Yes	Yes ❖	Yes
BTS Bioengineering	Freeemg	~USD 20.000	https://www.btsbioengineering.com/products/freeemg-surface-emg-semg/	No	No	No (Tethered)
Noraxon	Ultium EMG	~USD 20.000	https://www.noraxon.com/our-products/ultium-emg/#1541097720904-fe85b033-bd50	No	No	No
Delsys	Trigno	~USD 20.000	www.delsys.com	No	No	Yes
MyMyo Science	MyMyo	NA	http://mymyo.science/	Yes	Yes? 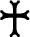	Yes
Cometa	PicoEmg	NA	https://www.cometasystems.com/products/picoemg	Yes	No	Yes
Myon	Aktos	NA	https://www.myon.ch/aktos	No	No	Yes

*NA, not available. ❖depends on the model. 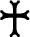
probably affordable, cost not available*.

We were pleased to find that at least four devices met all the predefined criteria, which provides the technical means to use sEMG directly in the office. This finding suggests that EMG device accessibility would not necessarily mean a barrier for the use of sEMG.

Finally, another issue could be related to the cost of electrodes, which may impose another barrier. Nevertheless, for most of the applications, either disposable (~U$0.15/piece) or reusable (~U$0.40/piece) electrodes do not constitute a substantial obstacle for using sEMG (reference prices obtained from amazon.com).

## Conclusions

Based on available information and personal insights, we have discussed some of the barriers to the use of sEMG that might be relevant in a developing country such as Chile. Several socioeconomic, and political aspects of our country determine that health policies favor those interventions that are highly cost-effective. The failure of the literature to translate into decisive outcomes has kept sEMG restricted mostly to research. To open a path for the inclusion of sEMG into clinical guidelines that recommend it as a necessity and not only as a complementary tool, it will be necessary to produce well designed and outcome-guided studies that also attain clinical standards. This will lead to results suitable for pooling into a meta-analysis, which may influence contexts that favor cost-effectiveness.

Who should generate this research? We think that this type of literature can arise more easily from transdisciplinary teams of clinical professionals (physicians, physical therapists, speech therapists, etc.) and developers (engineers, designers, etc.), in which the patients are at the very center of their activity. Clinicians may perfectly understand patients' problems, but without help from engineers, they will not be able to solve them. On the other hand, engineers and designers that are not connected to a clinical setting may create solutions that are either too complex or too difficult to implement and do not necessarily solve practical problems. In either case, the patients' particular needs are left unmet. We think that these types of interactions are probably the best remedy to transform problems and needs into meaningful solutions and to advance the research on sEMG.

In a different vein, our analysis of the curricula from Chilean PT schools, confirmed our suspicion of a lack of training in this area. In the schools with EMG training, this is taught mainly in one or two courses during the entire career, and the training takes place at the beginning of the career but not in the clinical internships, which may explain why PTs do not regularly use sEMG in their practice.

Regarding the accessibility of sEMG devices, we found that at least four devices met predefined criteria of being portable, affordable, and easy to operate. This suggests that accessibility or price does not constitute in itself a barrier for the use of sEMG in the clinical practice and that the main limitations arise from political and economic characteristics of the country, from a paucity of compelling clinical indications, and from an insufficient amount of training.

Finally, some of the barriers for the use of sEMG in the clinical domain may interact with each other in a circular manner, for example, the paucity of clinical evidence and the view of sEMG only as biofeedback may create insufficient pressure for both the development of health policies and for the training of rehabilitation professionals in sEMG. This insufficient training leads to an insufficient mass of professionals using sEMG, which leads to fewer grant applications and less research in the area, which leads us to the starting point. Also, there could be insufficient teaching dedicated to instrumentation or on technologies for rehabilitation. These types of courses could broaden the view on interventions and techniques that could enrich the clinical practice of rehabilitation professionals.

Therefore, we think there is a need for more high-quality clinical evidence that presents an unavoidable pressure to employ this technique. There is a need for advancing the training not only in PT schools, but also in speech therapy and occupational therapy as well. A critical mass of professionals trained on these techniques backed up by sufficient clinical evidence may create the perfect scenario for the massive use of surface electromyography.

## Data Availability Statement

Publicly available datasets were analyzed in this study. This data can be found here: https://data.worldbank.org/indicator/SI.POV.GINI,https://data.worldbank.org/indicator/SH.XPD.GHED.GD.ZS, https://www.mifuturo.cl/buscador-de-estadisticas-por-carrera.

## Author Contributions

JA-R and HM-V contributed equally to the manuscript and approved the submitted version. All authors contributed to the article and approved the submitted version.

## Conflict of Interest

The authors declare that the research was conducted in the absence of any commercial or financial relationships that could be construed as a potential conflict of interest.
